# Development of a reverse transcription recombinase polymerase amplification combined with lateral flow assay for equipment-free on-site field detection of tomato chlorotic spot virus

**DOI:** 10.1186/s12985-023-02097-w

**Published:** 2023-06-22

**Authors:** Salih Yilmaz, Ozgur Batuman

**Affiliations:** Department of Plant Pathology, Southwest Florida Research and Education Center, University of Florida, Immokalee, FL 34142 USA

**Keywords:** RT-RPA, Tospovirus, In-field detection, Equipment-free, Portable, Endonuclease IV (Nfo)

## Abstract

**Background:**

Tomato chlorotic spot virus (TCSV) is an economically important, thrips-transmitted, emerging member of the *Orthotospovirus* genus that causes significant yield loss mainly in tomatoes, but also in other vegetable and ornamental crops. Disease management of this pathogen is often challenging due to the limited availability of natural host resistance genes, the broad host range of TCSV, and the wide distribution of its thrips vector. Point-of-care detection of TCSV with a rapid, equipment-free, portable, sensitive, and species-specific diagnostic technique can provide prompt response outside the laboratory, which is critical for preventing disease progression and further spread of the pathogen. Current diagnostic techniques require either laboratory-dependent or portable electronic equipment and are relatively time-consuming and costly.

**Results:**

In this study, we developed a novel technique for reverse-transcription recombinase polymerase amplification combined with lateral flow assay (RT-RPA-LFA) to achieve a faster and equipment-free point-of-care detection of TCSV. The RPA reaction tubes containing crude RNA are incubated in the hand palm to obtain sufficient heat (∼36 °C) for the amplification without the need for equipment. Body-heat mediated RT-RPA-LFA is highly TCSV-specific with a detection limit as low as ∼6 pg/μl of total RNA from TCSV-infected tomato plants. The assay can be performed in 15 min in the field.

**Conclusion:**

To the best of our knowledge, this is the first equipment-free, body-heat-mediated RT-RPA-LFA technique developed to detect TCSV. Our new system offers a time-saving advantage for the sensitive and specific diagnostic of TCSV that local growers and small nurseries in low-resource settings can use without skilled personnel.

## Introduction

Tomato chlorotic spot virus (TCSV) is an emerging member of *Orthotospovirus*, a virus genus that economically impacts numerous vegetable and ornamental crops [1, 2]. The genome is composed of three ambisense segments of single-stranded RNAs known as small (S), medium (M), and large (L) [3]. TCSV is transmitted by thrips vectors, mainly *Frankliniella occidentalis* and *F. schultzei *[4]*.* The spread and severity of TCSV disease depend exclusively on plant susceptibility and thrips transmissibility. TCSV was first identified in tomatoes in the 1990s in Brazil [5] and Argentina [6]. The disease caused by TCSV was first reported in the USA in 2012 from Florida [7]. Shortly thereafter, disease outbreaks associated with TCSV were observed in the same region and caused economic losses in tomato production in south Florida. During later years, the incidence of TCSV in this region increased to a range of 30–40%, resulting in growers abandoning their fields due to the high incidence [8]. TCSV is considered an economically important emerging pathogen in Florida and predominantly occurs along with other known orthotospoviruses, including tomato spotted wilt virus (TSWV) and groundnut ring spot virus (GRSV) [9]. The virus was later reported in other parts of the continental United States (Ohio and New York) and several Caribbean islands (Puerto Rico, Dominican Republic, and Cuba) [10–14].

Rapid disease diagnosis, the use of resistant tomato cultivars (with the *Sw-5* gene), and thrips and weed control are fundamental steps of integrated pest management strategies used against orthotospoviruses [15–17]. Early diagnosis and exploring the genetic diversity of these pathogens are critical for disease eradication and preventing the further spread of the disease.

Correct field diagnosis of orthotospoviruses at the species level is difficult due to the symptom similarities of common orthotospoviruses such as TSWV, TCSV, and GRSV in most host plants [2, 7]. Several detection methods have been developed to detect TCSV, including enzyme-linked immunosorbent assay (ELISA), reverse transcription-polymerase chain reaction (RT-PCR), and reverse transcription-loop-mediated isothermal amplification (RT-LAMP) [2, 7, 18, 19]. These methods are often, require lengthy processes, special laboratory equipment, or are limited to only laboratory applications for diagnostics. Developing simple, rapid, and portable field diagnostic techniques for such diseases is imperative. Previously, we developed a field detection technique for TCSV using handwarmer-mediated RT-LAMP in under 35 min [19]. Alternative isothermal amplification methods with lower temperature dependency for exponential amplification have also been developed for rapid, sensitive, and inexpensive disease detection of other plant pathogens including but not limited to tomato mottle virus (ToMoV), tomato yellow leaf curl virus (TYLCV) and bacterial spot of tomato [20, 21].

Recombinase polymerase amplification (RPA) is a detection technology that requires constant low temperature (37–42 °C), unlike the higher temperature needed required for the LAMP assay (65 °C). Due to its simplicity, sensitivity, and cost efficiency, RPA has been widely used in the detection of various plant pathogens, including RNA viruses, in combination with reverse transcriptase enzymes [20, 22, 23]. RPA reaction utilizes three core enzymes: recombinase, single-stranded DNA-binding protein (SSB), and strand-displacing DNA polymerase. Initially, the recombinase enzyme binds to oligonucleotide primers in the presence of adenosine triphosphate (ATP) and a crowding agent (a high molecular weight polyethylene glycol), forming a recombinase-primer complex [24]. The complex then scans for a homologous sequence in the double-stranded DNA corresponding to each primer. The recombinase inserts the oligonucleotide primers at the complementary DNA site. The SSB proteins stabilize the displaced DNA to prevent the ejection of inserted primers via branch migration. Finally, the recombinase disassembles and leaves the 3′-end of the primer for a strand displacing DNA polymerase binding, which elongates the primer and initiates exponential amplification [21, 25].

The RPA can be further improved for extra functionality by the addition of different enzymes, including a reverse transcription for RNA detection, endonuclease IV for lateral flow assay (LFA), or exonuclease III for the real-time detection of fluorescent oligonucleotide probes [26, 27]. The assays can be performed within 20 min, and the results can be monitored in real-time or visualized either on agarose gel electrophoresis (AGE) or with LFA. Therefore, the RPA assay has great potential for in-field disease diagnostic applications.

In this study, we explored the use of reverse transcriptase (RT) and endonuclease IV (Nfo) enzymes in combination with RPA assay for TCSV detection. The RT enzyme allows the partial TCSV RNA genome sequence, specifically the *NSm* gene*,* to be synthesized to cDNA, which is simultaneously amplified via RPA. DNA repair enzyme (Nfo) is used to obtain amplification results with the universal LFA. Lateral flow assay is relatively easy, low-cost, and equipment-free [28, 29]. The RT-RPA-LFA assay utilizes two primers, including an unlabeled forward and 5′-Biotin labeled reverse primer, and a single probe with the 5′-FAM antigenic label, single tetrahydrofuran (THF) spacer at the middle, and a C3-spacer (polymerase extension blocking group) at its 3′-end [26]. The Nfo enzyme recognizes the mismatch created by THF residue and cuts the probe at that site. This creates a 5′-FAM labeled primer that is free from its blocking part and initiates the amplification with a smaller amplicon preference [30]. A final product of RPA dual-labeled with FAM and biotin can be detected using a lateral flow assay labeled with gold-labeled anti-FAM antibodies and anti-Biotin antibodies [31].

In this study, RT-RPA assay was developed for the first time for TCSV detection and adopted as an in-field diagnostic tool in combination with LFA. Moreover, our approach uses crude extracts for rapid RNA extraction and body heat to facilitate equipment-free RPA and LFA detection. This system can also occur without lab equipment and trained personnel for a convenient, in-field readout.

## Materials and methods

### Source of plant sample and virus maintenance

Tomato plants exhibiting chlorotic and necrotic leaf spot symptoms were collected from a field in Miami-Dade County (Homestead, Florida, USA; TCSV FL-HS21-S1 isolate) in 2021. Fresh symptomatic leaf and/or fruit pericarp tissues infected with TCSV (confirmed with RT-PCR) were mechanically inoculated in two to three weeks-old, five non-*Sw-5* tomato (cv. Florida 47) plants. Ice-cooled mortar and pestle were used to homogenize approximately 1 g of symptomatic plant tissue in 10 ml of 0.01 M phosphate buffer (pH 7), including 0.2% sodium sulfite and 0.01 M 2-mercaptoethanol [32]. Lightly carborundum-dusted leaves were rub-inoculated with a finger after being dipped in the inoculum. Inoculated plants were maintained in the greenhouse, and TCSV infection was confirmed. These plants were then used as virus inoculum sources for further RNA extraction using conventional and rapid methods described below.

### Total RNA and crude RNA extractions

Total RNA was extracted from symptomatic, two field-collected tomatoes and/or five mechanically inoculated inoculum source plants from the greenhouse using the Quick-RNA MiniPrep kit (Zymo Research, Irvine, CA, USA) according to the manufacturer's instructions. RNA obtained by conventional procedure was maintained at −80 °C until needed. Crude RNA was extracted from the same inoculum sources using a rapid (under a minute) nucleic acid extraction method combined with homemade individual absorption strips as previously described [19, 33] and used immediately in the assays.

### Conventional RT-PCR assay

TCSV RNA (0.6 μg/μl) was subjected to cDNA synthesis using the Superscript II (200 U/μl) reverse transcriptase (Invitrogen, Carlsbad, CA, USA). cDNA was then used as a template for PCR assays using the same forward and reverse primers that are newly designed for RPA assay in this study (Table 1). A total of 25 µl of PCR reaction mix contained 12.5 µl DreamTaq Green PCR Master Mix (Thermo Fisher Scientific, Waltham, MA, USA), 1 µl each forward and reverse primers (10 µM), 2 µl of cDNA, and 8.5 µl nuclease-free water. Thermocycler settings for the PCR amplification consist of 2 min denaturation at 94 °C, followed by 30 cycles of 94 °C for 20 s, 60 °C for 20 s, and 72 °C for 20 min, with a final extension at 72 °C for 7 min. For the confirmation of the presence or absence of other tomato infecting viruses, including representative members of Orthotospovirus (TSWV), Ilarvirus (tomato necrotic streak virus; TomNSV), Cucumovirus (cucumber mosaic virus; CMV), Tobamovirus (tomato brown rugose fruit virus; ToBFRV, tomato mosaic virus; ToMV), Potexvirus (pepino mosaic virus; PepMV), Amalgavirus (southern tomato virus; STV), and Begomovirus (TYLCV) specific primer pairs for each virus were used in separate reactions [19, 34–40]. PCR products were visualized on 1% agarose gels with Apex™ Safe DNA Stain (Genesee Scientific, San Diego, CA, USA).

**Table 1:** Names, nucleotide sequences and genomic characteristics of RT-RPA-primers and -probe designed to target *NSm* gene and used in this study for tomato chlorotic spot virus (TCSV) detection

Primer/probe name	Sequence (5′–3′)	Length (nt)	GC (%) content	Genome position^a^
TCSV_RPA_NSm_F	ATTAATTGACCCTAACATGCCTTCTGACAAGC	32	40.6	414–445
TCSV_RPA_NSm_R	^b^5Biosg/GCAACACTTATCTTTATCGG CTCTTGGAGAATC	33	42.4	666–634
TCSV_RPA_NSm_Probe	^c^6-FAM/ATCTAAACTGGTCTATCCCA AAAGCGAACAA/^d^idsp /ACACCTG AAAACTGCTG/^e^3SpC3	49	42.9	500–530/532–548

^a^Genome position based on the nucleotide sequence of TCSV (GenBank Accession No. ON783718)

^b^5Biosg, Biotin

^c^6-FAM, 6-carboxyfluorescein (Fluorescein)

^d^idsp, internal abasic nucleotide analogue (THF)

^e^3SpC3, Spacer (polymerase extension blocking group)

### RPA primer and probe design for TCSV detection

**Fig. 1:**
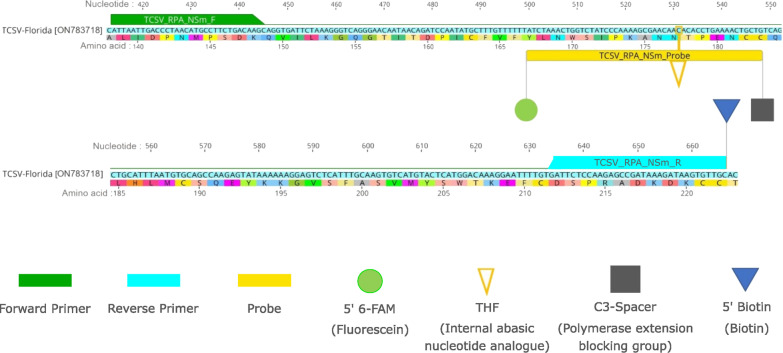
Targeted *NSm* gene sequence of tomato chlorotic spot virus (TCSV) (GenBank: ON783718) and demonstration of sequences and postions of the RPA primers and probe used in the RT-RPA assay

The conserved sequence region from the viral movement protein (*NSm*) of TCSV isolates, but with divergence from representative other orthotospovirus species, was targeted for RPA primers and probe design. The available nucleotide sequences of the *NSm* gene of TCSV (GenBank Accession No. JX244200; KX463273; KY820957; MH427862; ON783718) and other orthotospovirus species, including GRSV, TSWV, and impatiens necrotic spotted wilt virus (INSV) (GenBank accession no. HQ644141; MK524206; KT972591) from the National Center for Biotechnology Information database (NCBI) were obtained and aligned, and sequence homology was analyzed by using Geneious prime version 2022.0.2. Primers and probe were designed based on guidelines of TwistAmp™ (TwistDx, Cambridge, UK) and AmplifyRP Acceler8 (Agdia, Inc., Elkhart, IN, USA) RPA kit manual. Forward and reverse primers were designed to be between 30–35 nucleotide (nt) in length. The 5′-end of the reverse primer was labeled with biotin, while the forward primer was kept unlabeled. The probe was designed to be 46–52 nt in length, and located between the forward and reverse primer. At least 30 nt of the probe was located in between the 5′-end of the tetrahydrofuran (THF) site and at least 15 nt of the remaining sequence was placed in between the THF site and the 3′-end (Fig. 1). The RPA probe was labeled at the 5′-end with ﻿6-carboxy-fluorescein (FAM), the 3′-end was ﻿blocked with a C3-spacer, and the 32nd base of the probe was replaced with THF. The primers and probe were synthesized by the Integrated DNA Technologies, Inc. (IDT, Coralville, IA, USA).

### RT-RPA primer optimization

RT-RPA was first performed using the TwistAmp® Basic Kit (TwistDx, Cambridge, UK) in a reaction volume of 50 µl following the manufacturer’s instructions with minor modifications. According to the manufacturers instructions, a 47.5 µl reaction mix was prepared by adding 2.4 µl of each primer (10 mM), 29.5 µl rehydration buffer, 0.5 µl Superscript II (200 U/μl) reverse transcriptase (Invitrogen, Carlsbad, CA, USA), and 10.7 µl nuclease-free water. The reaction mixture was then mixed with freeze-dried pellets in reaction tubes provided by TwistAmp Basic. Normally this reaction would contain 1 µL RNA and 2.5 µL of magnesium acetate, however, in order to increase the number of samples we could process with the kit, we divided the 47.5 µl reaction into two 22.75 µL reactions. We subsequently added 1µl RNA from two different tomato plants infected with TCSV and 1.25 mL of 280 mM magnesium acetate. In total, 25 μl of each reaction mixture was gently mixed by pipetting and briefly centrifuged. In the initial primer optimization step, the reaction tubes were incubated at 40 °C for 30 min using thermocycler following the manufacturer's instructions, without the use of the probe, to obtain clear results with agarose gel electrophoresis. The primers used in this assay are listed in Table 1. After the initial verification of RT-RPA primers, to assess optimum reaction temperature, the RPA reaction tubes were then incubated at 30, 33, 35, 36, 37, 38, 39, 40, and 45 °C in the thermocycler for 30 min. After the optimal temperature was defined, different incubation times (5, 10, 15, 20, 25, 30, 35, and 40 min) were evaluated to determine optimal reaction durations for TCSV RT-RPA detection. Reaction tubes were then incubated at 95 °C for 5 min to ensure the termination of enzymatic activity. RPA amplicons for each reaction were purified using DNA Clean & Concentrator™ 100 kit (Zymo Research, Irvine, CA, USA). Purified DNA products were visualized on 1% agarose gels with Apex™ Safe DNA Stain (Genesee Scientific, San Diego, CA, USA). The same methods were followed in all RPA assays for agarose gel electrophoresis throughout the study.

### RT-RPA primer sensitivity and specificity

To assess the sensitivity of RT-RPA assay and compare it with RT-PCR, tenfold serial dilutions (from 1 × 10^–0^ to 1 × 10^–9^) of TCSV-RNA extracts were prepared in nuclease-free water and used in cDNA synthesis. These cDNAs were then subjected to RT-RPA and RT-PCR analysis, respectively. To assess the specificity of the TCSV RT-RPA assay, common tomato infecting viruses, including representative members of Orthotospovirus (TSWV), Ilarvirus (TomNSV), Cucumovirus (CMV), Tobamovirus (ToBRV and ToMV), Potexvirus (PepMV), Amalgavirus (STV), and Begomovirus (TYLCV) were evaluated for cross-reactivity with RT-RPA primers designed specific for TCSV NSm in this study. To ensure the consistency of the test, specificity and sensitivity assays were repeated three times with appropriate controls for each virus-species tested.

### Development of RT-RPA- lateral flow assay (RT-RPA-LFA) and assessment of its specificity and sensitivity

To eliminate the agarose gel electrophoresis step, LFA was used following the RT-RPA amplification, including the probe. RT-RPA-LFA was performed using AmplifyRP Acceler8 ﻿(Agdia, Inc., Elkhart, IN, USA) in a reaction volume of 10 µl following the manufacturer’s instructions with minor modifications. In total, 8.5 µl of reaction mix was prepared by adding 0.42 µl of each primer (10 µM), 5.9 µl rehydration buffer, 0.12 µl of probe (10 µM), 0.25 µl Superscript II (200 U/µl) reverse transcriptase (Invitrogen, Carlsbad, CA, USA), and 1.39 µl nuclease-free water. The reaction mixture was then mixed with freeze-dried pellets in reaction tubes provided by AmplifyRP Acceler8. The total RNA (1 μl) or crude RNA extract of TCSV infected tomato plant and 280 mM magnesium acetate (0.50 μl) were added to the 8.5 μl reaction mix. In total, 10 μl of the reaction mixture was gently mixed by pipetting and briefly centrifuged and incubated at 36 °C for 20 min. After the RT-RPA assay, the amplification product was immediately tested on lateral flow assays (LFA) using two different universal LFAs for the detection of biotin- and FITC-labeled analytes, Agdia_AmplifyRP® amplicon detection chamber (Agdia, Inc., Elkhart, IN, USA) and Milenia ® HybriDetect lateral flow test strips (Milenia Biotec, Gießen, Germany). A 5 μl of RT-RPA amplification product pipetted directly on the sample application area in Milenia ® HybriDetect lateral flow test strip. The test strips were then placed in a tube containing 100 μL HybriDetect Assay Buffer and waited for 5 min for the results. To test the same RT-RPA amplification product in other LFA test strips, a tube containing 5 μl of remaining RT-RPA amplification product was placed in Agdia_AmplifyRP® amplicon detection chamber according to the manufacturer’s instructions and waited for 5 min for results. The specificity of the RT-RPA-LFA was also evaluated against the above-mentioned tomato-infecting viruses. To determine the sensitivity of RT-RPA-LFA, the experiments were conducted involving tenfold serial dilutions ranging from 1 × 10^–0^ to 1 × 10^–9^ of TCSV RNA (0.6 µg/μl) and crude RNA extracts as templates. The performance of RT-RPA-LFA using tenfold serial dilutions of total RNA were compared with that of RT-PCR. Additionally, the performance of tenfold serial dilutions of crude RNA extracts were compared with a previously developed field-deployable RT-LAMP assay [19]. To generate a range of concentrations for the crude RNA, TCSV-infected crude RNA diluted with healthy tomato crude RNA extract. Field-portable RT-LAMP assay was performed following the protocol as described in Yilmaz et al. [19]. Lastly, specificity and sensitivity assays were repeated under the same conditions described above.

### Development and optimization of equipment-free RT-RPA-LFA using hand palm heat

To simulate and evaluate the on-field applicability of the RT-RPA-LFA to TCSV detection, crude RNA extracts from TCSV-infected fresh tomato leaves were prepared with the homemade individual absorption strips as described in Yilmaz et al. [19]. Absorption strips with crude RNA were dipped into the reaction tubes several times to release the templates. These reaction tubes were closed and incubated in the hand palm to obtain body heat (∼36 °C) for the amplification and without the need for equipment. The RT-RPA were incubated at 5, 10, 15, and 20 min in the hand palm to determine the lowest incubation time needed for amplification. The RT-RPA results were visualized using LFAs immediately after the incubation period. Once the incubation time was determined, the specificity of the RT-RPA-LFA was re-evaluated using crude extracts from freshly collected two different TCSV-infected and previously frozen TSWV-, ToBRFV-, TomNSV-infected, and healthy tomato tissues. The assay was repeated and tested on two different LFAs (with and without a chamber) to ensure the suitability of the test.

## Results

### RT-RPA primer evaluation and assay optimization

**Fig. 2:**
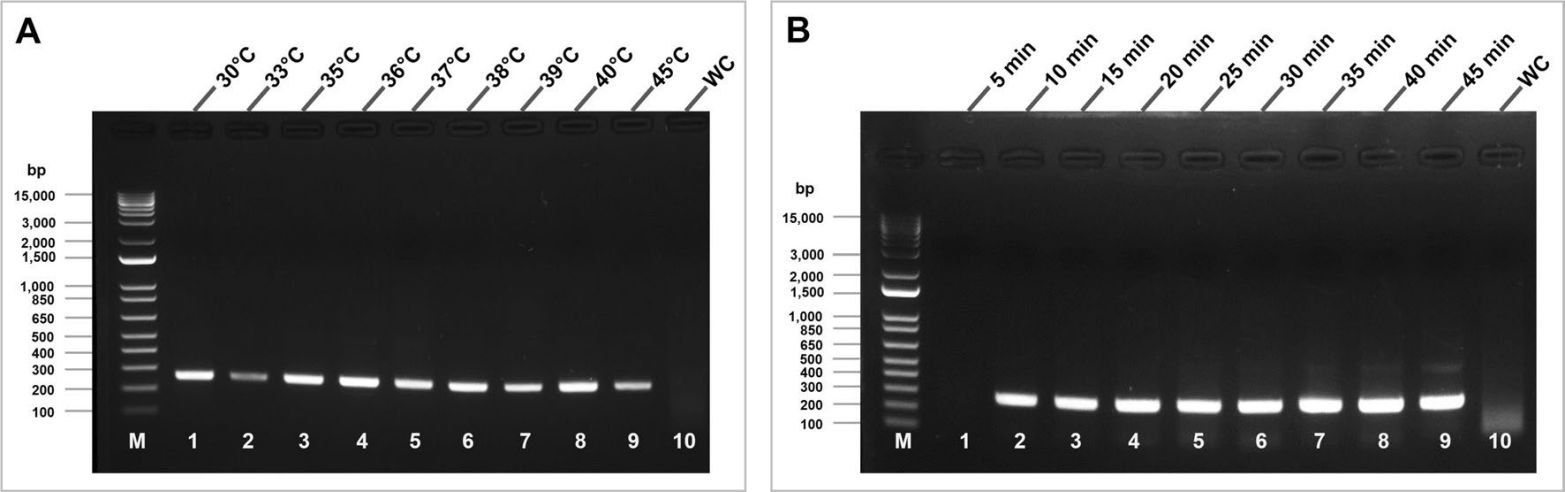
Optimization of RT-RPA reaction conditions for the detection of tomato chlorotic spot virus (TCSV) that visualized by agarose gel electrophoresis. **A** Effects of incubation temperatures on the RT-RPA reaction: lanes 1–9 with TCSV RNA template at 30, 33, 35, 36, 37, 38, 39, 40, and 45 °C for 30 min, respectively; lane 10, no-template (water) control at 36 °C; M, 1 kb DNA ladder. **B** Effects of incubation period on the RT-RPA reaction at 36 °C: lanes 1–9 with TCSV RNA template for 5, 10, 15, 20, 25, 30, 35, 40, and 45 min, respectively; lane 10, no-template (water) control (WC) for 45 min; lane M, 1 kb DNA ladder

The agarose gel electrophoresis (AGE) results of RT-RPA showed that the DNA was amplified successfully in each tested incubation temperature, but stronger bands were seen with the incubation temperatures of 35–40 °C (Fig. 2A). Since 36 °C was the closest temperature tested in this study to average body temperature (~ 36.5 °C), this temperature was selected for further assays. Different incubation times (5, 10, 15, 20, 25, 30, 35, and 40 min) were then evaluated at 36 °C to determine the optimal reaction duration for RT-RPA detection of TCSV. The designed RPA primers were able to detect TCSV within 10 min from the start of the reaction. After 30 min of incubation, an additional faint nonspecific larger band (∼500 bp) appeared and became more visible with the increased incubation periods (Fig. 2B). Therefore, the reaction conditions of 10 to 20 min at 36 °C were selected for optimum detection of TCSV using the RPA primers TCSV_RPA_NSm_F and TCSV_RPA_NSm_R.

### RT-RPA Sensitivity and Specificity

**Fig. 3:**
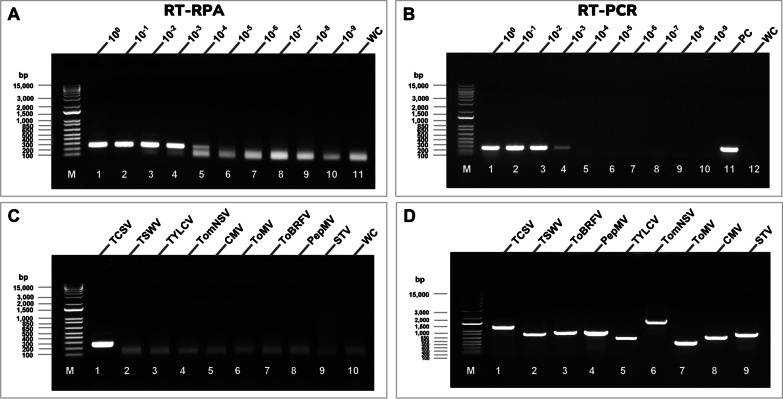
Comparison of specificity and sensitivity of RT-RPA and RT-PCR assays for detection of tomato chlorotic spot virus (TCSV) by agarose gel electrophoresis. **A** Sensitivity of RT-RPA assay for detection of TCSV. Lane 1, total RNA of TCSV (0.6 ug/μl); lanes 2–10, tenfold serial dilutions of total RNA; lane 11, no-template (water) control; M, 1 kb DNA ladder. **B** Sensitivity of RT-PCR assay for detecting TCSV using the same RPA primers used in RPA assay in A. Lane 1, total RNA of TCSV (0,6 μg/μl); lanes 2–10, tenfold serial dilutions of total RNA; lane 11, positive control (PC); lane 12, no-template (water) control (WC); M, 1 kb DNA ladder. **C** Detection specificity of TCSV-specific RT-RPA assay, and **D** virus specific RT-PCR assays. Lane 1, TCSV; lane 2, tomato spotted wilt virus (TSWV); lane 3, tomato brown rugose fruit virus (ToBRFV); lane 4, pepino mosaic virus (PepMV); lane 5, tomato yellow leaf curl virus (TYLCV); lane 6, tomato necrotic streak virus (TomNSV); lane 7, tomato mosaic virus (ToMV); lane 8, cucumber mosaic virus (CMV); lane 9, southern tomato virus (STV); M, 1 kb DNA ladder

The sensitivity of RT-RPA and RT-PCR was evaluated and compared by using tenfold serial dilutions (from 1 × 10^–0^ to 1 × 10^–9^) of TCSV RNA extracts with nuclease-free water. Sharp bands were observed in RT-RPA assays with the dilutions from 1 × 10^–0^ (0.6 µg/μl) to 1 × 10^–3^ (600 pg/μl) in AGE (Fig. 3A). At 60 pg/μl (10^–4^ dilution), the positive band was weaker, yet remained distinguishable. Compared to RPA, the AGE result of RT-PCR amplification using RNA at the 10^–3^ dilution showed a substantially faint band (Fig. 3B). At 10^–4^, RT-PCR was unable to detect the TCSV RNA. Therefore RT-RPA (10^–4^) assay was ten times more sensitive than RT-PCR (10^–3^) based on the results visualized on the AGE.

The specificity of the RT-RPA assay was evaluated and determined by using RNAs and DNA (for TYLCV) obtained from common tomato-infecting viruses, including representative members of Orthotospovirus (TSWV), Ilarvirus (TomNSV), Cucumovirus (CMV), Tobamovirus (ToBRV, ToMV), Potexvirus (PepMV), Amalgavirus (STV), and Begomovirus (TYLCV). RT-RPA amplification was observed only with the TCSV-infected sample, confirming the TCSV-specificity of the RT-RPA assay (Fig. 3C). RT-PCR confirmed the presence of other viruses with virus species-specific primers, and results were visualized on AGE (Fig. 3D).

### RT-RPA combined with lateral flow assay (LFA) for on-site detection of TCSV

**Fig. 4:**
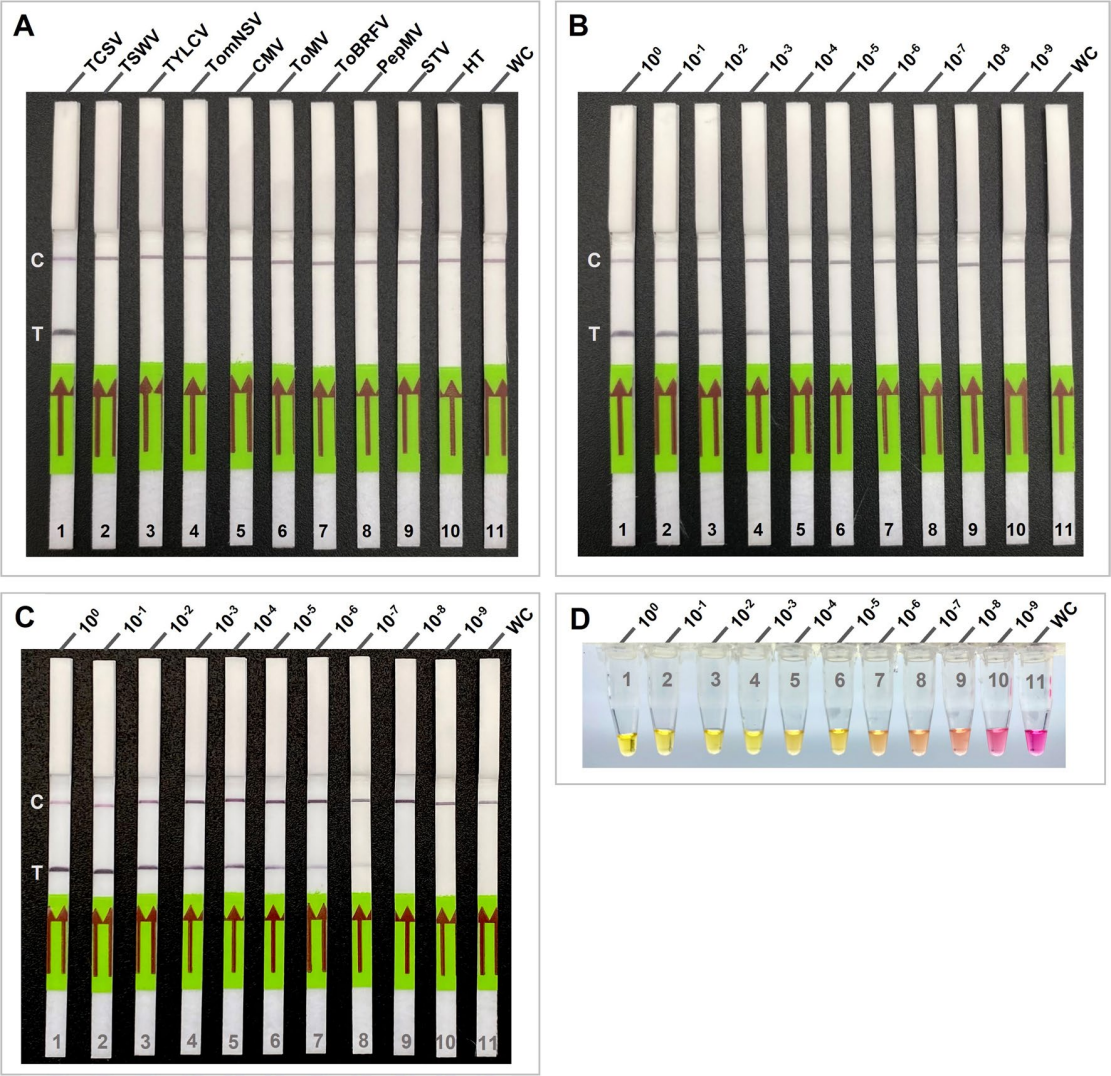
Specificity and sensitivity of lateral flow assay (LFA) combined with RT-RPA, and its comparison with RT-LAMP assay. **A** Specificity of RT-RPA-LFA assay used for testing tomato chlorotic spot virus (TCSV) and other tomato infecting viruses. Lateral flow test strip 1, TCSV; 2, tomato spotted wilt virus (TSWV); 3, tomato yellow leaf curl virus (TYLCV); 4, tomato necrotic streak virus (TomNSV); 5, cucumber mosaic virus (CMV); 6, tomato mosaic virus (ToMV); 7, tomato brown rugose fruit virus (ToBRFV); 8, pepino mosaic virus (PepMV); 9, southern tomato virus (STV); 10, healthy tomato (HT); 11, no-template (water) control (WC). **B** Sensitivity of RT-RPA-LFA in detecting TCSV. Lateral flow test strip 1, total RNA of TCSV (0,6 ug/μl); lanes 2–10, tenfold serial dilutions of total RNA; lane 11, no-template (water) control (WC). **C** Sensitivity of RT-RPA-LFA in detecting TCSV. Lateral flow test strip 1, crude RNA of TCSV; lanes 2–10, tenfold serial dilutions of crude RNA; lane 11, no-template (water) control (WC). C, control line; T, test line. **D** Sensitivity of RT-LAMP assay in detecting TCSV using colorimetric endpoint detection with pH indicator (phenol red). Tube one crude RNA of TCSV; tubes 2–10, tenfold serial dilutions of crude RNA; tube 11, no-template (water) control (WC)

An LFA was used to speed up the detection process and eliminate laboratory-dependent RPA visualization methods (i.e., AGE; agarose gel electrophoresis). Here, the specificity of RT-RPA-LFA was assessed using the TCSV and several other tomato-infecting viruses as demonstrated in the above specificity assays. The specificity assay showed that RT-RPA combined with LFA reacted only to RNA extracts from TCSV-infected tomato samples. No cross-reaction was obtained with RNA or DNA (for TLYCV) extracts from any of the eight other tomato-infecting viruses tested, including TSWV, TYLCV, TomNSV, CMV, ToMV, ToBRFV, PepMV, STV, and healthy tomato (Fig. 4A). The results confirmed that RT-RPA, in combination with LFA, remains virus-specific and detects only TCSV.

To evaluate the sensitivity of the RT-RPA-LFA detection, 10^0^ to 10^–9^ dilutions of total RNA samples and crude RNA extracts of TCSV were used as templates. RT-RPA-LFA detected TCSV with 6 pg/μl (10^–5^ of dilution) of total RNA, whereas agarose gel electrophoresis-based RT-RPA and RT-PCR detected with 60 pg/μl (10^–4^ of dilution) and 600 pg/μl (10^–3^ of dilution) of RNA, respectively (Fig. 4A, B). These results indicated that the detection limit for RT-RPA-LFA is 100 times higher than the RT-PCR and ten times higher than the RT-RPA-agarose gel assay. RT-RPA-LFA further compared with previously developed in-field RT-LAMP assay [19] using 10^0^ to 10^–9^ dilutions of crude RNA extract (Fig. 4C, D). The detection limit of RT-LAMP was found to be as low as 10^–8^ dilution whereas in RT-RPA-LFA faint band observed in 10^–7^ dilution, suggesting RT-LAMP is ten time more sensitive than RT-RPA-LFA in field conditions.

### Validation of equipment-free RT-RPA-LFA assay in the field

**Fig. 5:**
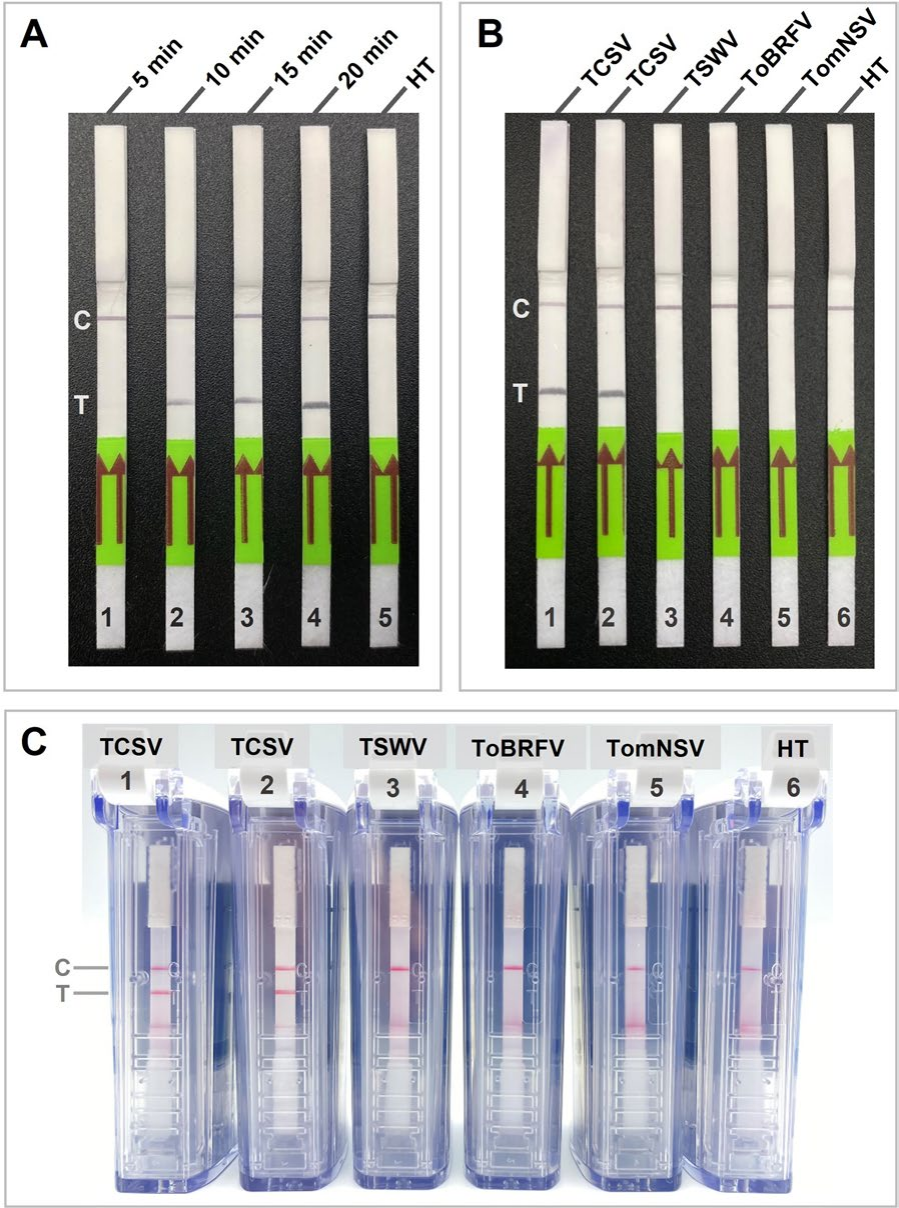
Lateral flow assay (LFA) detections of body heat-mediated RT-RPA assay **A** Effects of incubation periods on the RT-RPA-LFA detection at 36 °C: lateral flow strip 1–4 with tomato chlorotic spot virus (TCSV) template for 5, 10, 15, and 20 min, respectively; 5-healthy tomato for 15 min. RT-RPA cross-reactivity test using crude extract of symptomatic tomato plants visualized by using **B** universal lateral flow strips and **C** LFA detection chambers: lateral flow strip 1 and 2, TCSV; 3, tomato spotted wilt virus (TSWV); 4, tomato brown rugose fruit virus (ToBRFV); 5 tomato necrotic streak virus (TomNSV); 6, healthy tomato-control (HT). C: control line; T: test line

To achieve a portable and equipment-free detection method for TCSV in the field, crude extracts of freshly collected and mechanically inoculated symptomatic and healthy tomato plants were used as a template for RT-RPA-LFA. Crude extracts were obtained under a minute using individual absorption strips and immediately transferred to RT-RPA reaction tubes. Body-heat-mediated RT-RPA-LFA using hand warmth was first validated with various incubation periods (5, 10, 15, and 20 min). The LFA results indicated that the body-heat-mediated RT-RPA was able to detect TCSV from freshly collected tomato leaves in as little as 10 min (Fig. 5A). However, as the incubation period increased to 15 or 20 min, the intensity of positive bands on lateral flow strips also increased and became more visible (Fig. 5A). Therefore, we recommend about 15 min incubation period for equipment-free RT-RPA-LFA for more reliable results in field conditions.

The cross-reactivity of equipment-free RT-RPA-LFA was re-evaluated this time by using crude extracts instead of total RNAs from symptomatic tomato plants infected with TSWV, ToBRFV, or TomNSV (RT-PCR verified; Fig. 3D). The RT-RPA assay was repeated, and the results were visualized using two different types of commercially available LFAs (Fig. 5B, C). Both types of assays evaluated here gave consistent and comparable results. The body-heat mediated RT-RPA-LFA was also confirmed to be specific only to TCSV in both types of lateral flow assays tested (Fig. 5B, C).

## Discussion

TCSV is an economically important emerging pathogen that infects mainly tomatoes, but also other vegetable and ornamental crops. Simple, rapid, and sensitive field-detection of TCSV is critical for preventing disease progression and further virus spread within and between tomato fields. Previously, a laboratory-based and a field-deployable diagnostic methods was developed for detecting TCSV using hand-warmer-mediated RT-LAMP [19]. Despite its advantages, which are inexpensive, portable, and highly specific, the LAMP assay has a few limitations, including the need for a heating device and the inadequacy of downstream applications. Unlike LAMP, RPA assay products can be used for genetic diversity, phylogenetics, and further molecular studies, including cloning, direct sequencing, and restriction analysis [41–43]. Additionally, LAMP requires a larger set of primers, a higher temperature (65 °C), and a longer incubation time. In contrast, RT-RPA-LFA is relatively simple and can be performed with one set of primers and one probe in a lower constant temperature (38–42 °C), and a shorter incubation time [44]. RPA has significant advantages over PCR and RT-PCR assays commonly used in plant diagnostic laboratories. PCR techniques have high start-up costs, such as thermocycler instruments. RPA offers cheaper, significantly faster, and operationally simple advantages to diagnostic facilities without sacrificing specificity and downstream application capability [41]. Owing to these advantages, the RPA is becoming a more popular detection technique, including the diagnosis of tomato-infecting viruses and viroid, such as TSWV, begomoviruses (bean golden yellow mosaic virus (BGYMV), ToMoV and TYLCV), and tomato chlorotic dwarf viroid (TCDVd) [22, 23, 41, 45].

In this study, we developed a novel, body-heat mediated RT-RPA for faster and equipment-free detection of TCSV. Moreover, the newly developed RT-RPA showed no cross-reaction with RNA extracts from any of the other tomato extracts infected by various tomato-infecting viruses or DNA extract from TYLCV-infected tomato (Fig. 3). Thus, our RT-RPA assay was specific for the detection of TCSV.

To achieve complete field portability and avoid equipment dependence of the assay, the RT-RPA was further combined with a lateral flow assay (LFA), and the amplification was initiated by using body heat (i.e., warmth of hand palm). RT-RPA-LFA based detection was 100 times more sensitive than the RT-PCR and ten times more sensitive than agarose gel electrophoresis-based detection of RT-RPA (Figs. 4, 5). Superior sensitivity of LFA-based detections over AGE-based detections was previously reported in the other pathogen detections combined with RPA, LAMP, and PCR techniques [46–48]. To evaluate the sensitivity of in-field TCSV detection techniques, we used tenfold serial dilution of crude RNA extracts and compared previously developed RT-LAMP [19] and RT-RPA-LFA. RT-RPA-LFA was found to exhibit a detection limit with a faint band observed at the 10^–7^ dilution, while RT-LAMP demonstrated a lower limit of detection at the 10^–8^ dilution (Fig. 4C, D). These results suggest that, in the context of field conditions, RT-LAMP may be ten times more sensitive than RT-RPA-LFA. As expected, our newly developed RT-RPA-LFA showed no cross-reaction with RNA extracts from any of the other tomato extracts that were infected by various tomato infecting viruses or DNA extract from TYLCV-infected tomato that were tested in this study (Fig. 5).

Lastly, to simulate and validate field-applicability of equipment-free RT-RPA-LFA, crude extracts of field-collected and mechanically inoculated fresh tissue samples from TCSV-infected plants and previously frozen (at −80 °C) TSWV-, ToBRFV-, TomNSV-infected tomato leaf samples were used as a template. Here, crude extracts from TCSV-infected tomato samples were obtained in less than a minute in the field and used as a template for body-heat mediated RT-RPA-LFA. RPA is known to tolerate inhibitors present in crude samples, allowing for rapid on-site RNA detection [49, 50]. The RT-RPA assay was repeated and tested with two commercially available different lateral flow assays. The RT-RPA-LFA showed consistent and comparable results in both LFAs with and without a chamber (Fig. 5). To avoid carryover contamination, tubes containing post-amplification products should always be maintained closed, particularly in laboratory settings. In the case of using non-chambered LFAs, it is strongly recommended to perform the assay in different rooms or locations (e.g., in-field detection) during pre- and post-amplification to minimize the risk of contamination. Therefore, to avoid carryover contamination, it is recommended to use LFAs with chambers in enclosed spaces since they eliminate aerosolization of the post-amplification products to the test area [51–53]. However, universal LFAs without chambers are relatively lower cost, thus, we cautiously believe that they are more appropriate for tests conducted in open spaces such as in field conditions.

To the best of our knowledge, the equipment-free RT-RPA-LFA developed in this study is the first that is adopted to specifically detect TCSV, both in the laboratory and in-field. For the field-deployable application, the RT-RPA assay was combined with LFA detection, and the reaction was carried out by using crude RNA extract and body heat (hand palm), thus eliminating the need for any equipment. Once the equipment-free RT-RPA-LFA is performed, the results can be obtained in about 15 min in the field. The assay offers substantial advantages over current diagnostic methods for TCSV detection in both laboratory and in-field settings. Although the cost of the RT-RPA-LFA is estimated at $10.99 per sample, which is higher than previously developed field deployable RT-LAMP (estimated at $2.22) [19], the assay offers a faster and equipment-free alternative. This also makes it an ideal tool for rapid disease detection in any diagnostic setting, including in the field, nursery, or in areas without essential infrastructure.

Another inexpensive option for field detection of orthotospoviruses is a diagnostic test strip assay from two different companies, which is estimated at $6.2 or $3.05 per sample. However, this assay shows cross-reaction with TSWV, TCSV, and GRSV, thus making it impossible to distinguish TCSV without further molecular tests [54]. Finally, with its ability to support downstream applications, the RT-RPA-LFA is a much less labor-intensive, cheaper, and faster alternative to RT-PCR (estimated at $13.37 per sample) in diagnostic services and molecular laboratories.

In conclusion, due to its simplicity, ease of sample preparation, and equipment-free nature, the method developed in this study will benefit growers, as well as diagnosticians lacking proper infrastructure. Our RT-RPA-LFA is a rapid, inexpensive, virus-specific, and highly sensitive diagnostic technique for faster and early diagnosis of TCSV, thereby can be used as a tool for preventing further spread of the virus and subsequent disease outbreaks.

## Data Availability

All data and materials are disclosed to manuscript. Further information on data and materials can be requested from corresponding author.
